# Difficult Preoperative Diagnosis of Suspected Metal Hypersensitivity in a Case with Early Failure of Bipolar Hemiarthroplasty

**DOI:** 10.1155/2023/8656265

**Published:** 2023-05-31

**Authors:** Airi Shimmyo, Yu Takeda, Shigeo Fukunishi

**Affiliations:** ^1^Department of Orthopedic Surgery, Hyogo Medical University, Nishinomiya, Hyogo, Japan; ^2^Nishinomiya Kaisei Hospital, Nishinomiya, Hyogo, Japan

## Abstract

**Background:**

Metal hypersensitivity is a rare complication after total hip arthroplasty (THA), and no reliable diagnostic method for metal hypersensitivity to orthopedic metal implants has yet been established. *Case report*. A 57-year-old woman underwent hemiarthroplasty using a metal implant despite a skin allergy to metal jewelry. Two years after surgery, the patient developed early hemiarthroplasty failure and refractory erythema. Although the patient was clinically suspected to have a hypersensitivity to metal, the preoperative screening test was negative, and patient underwent revision surgery with cemented THA. Postoperatively, the erythema as well as her hip pain disappeared completely.

**Conclusion:**

Patients with clinically suspected metal hypersensitivity should undergo primary and revision total hip arthroplasty using hypoallergenic implants regardless of preoperative screening results.

## 1. Introduction

Metal hypersensitivity is a rare complication that can lead to the functional impairment and aseptic loosening after total hip arthroplasty (THA) [1, 2]. Metal hypersensitivity after total joint replacement is defined as a Type IV delayed-hypersensitivity allergic reaction, and although the exact cause is still unknown, it has been suggested that metal ions are released from the implants that trigger an immune response [3–6]. An accurate diagnostic test for metal hypersensitivity before and after total joint arthroplasty has not been established. Patch tests and lymphocyte transformation tests are commonly used, however, neither test leads to a definitive diagnosis. In this report, we present a case in which metal hypersensitivity was suspected as the cause of early failure of bipolar hemiarthroplasty (BHA), making correct preoperative diagnosis before revision THA difficult.

## 2. Case Report

**Figure 1:**
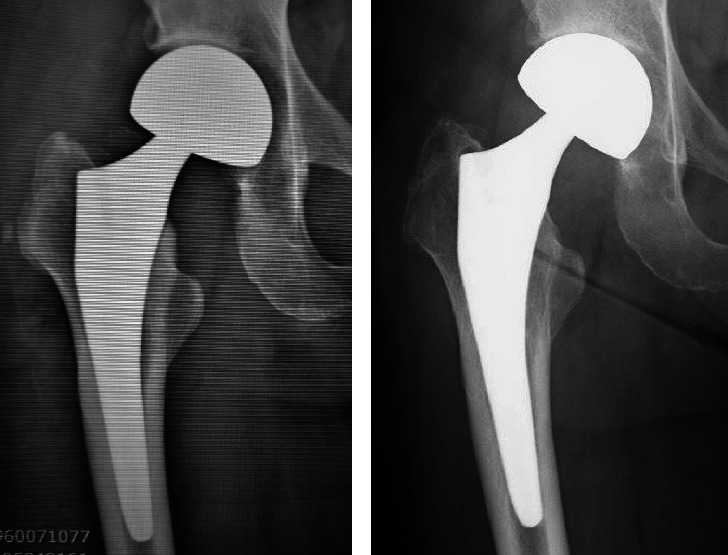
Plain radiograph of the right hip. (a) After initial surgery with BHA. (b) Two years after initial BHA.

**Figure 2:**
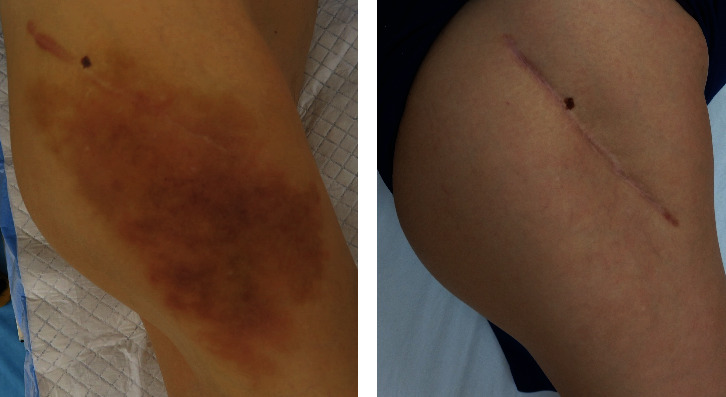
Gross findings of right hip. (a) Before revision surgery. (b) 3 months after revision surgery.

**Figure 3:**
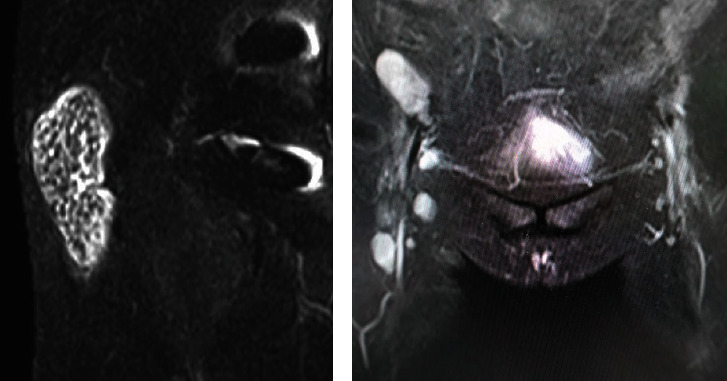
MRI. (a) Small honeycomb-shaped bodies within the cystic lesion on the T2-weighted STIR sequence. (b) Numerous inguinal lymphadenopathies on the T2-weighted STIR sequence.

**Figure 4:**
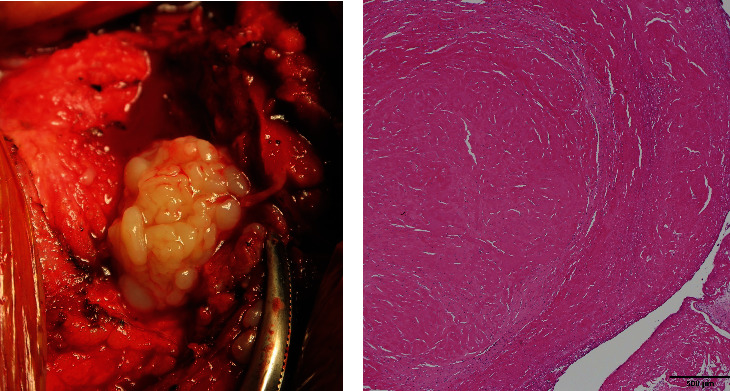
(a) Gross finding of rice body cyst. (b) Histological findings of the rice body: rice bodies were mostly composed of fibrin. Neutrophils, lymphocytes, or eosinophils were not recognized in the tissue.

**Figure 5:**
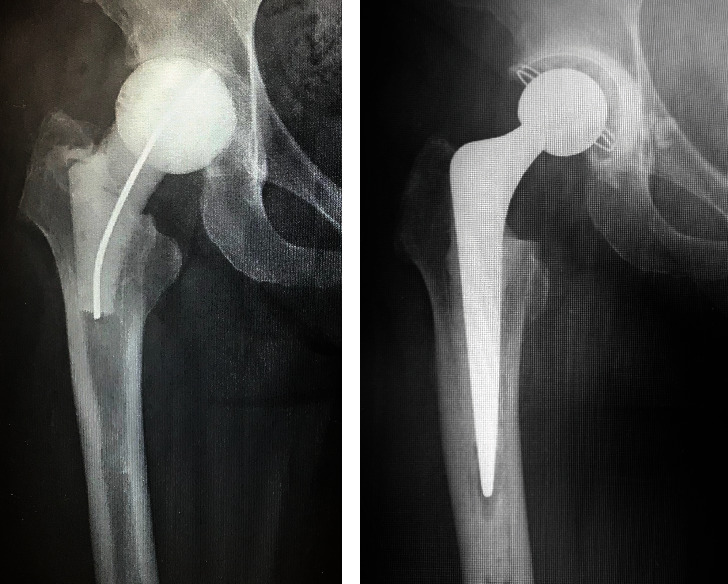
Postoperative plain radiograph. (a) Antibiotic cement mold was inserted in first surgery. (b) Cemented THA was performed in second surgery.

A 57-year-old woman sustained a femoral neck fracture of the right hip due to traffic trauma. The patient underwent BHA using a metal implant for a femoral neck fracture despite a history of hay fever, mild atopic dermatitis, and a skin allergy to metal jewelry. The prosthesis used during the hemiarthroplasty was a cobalt–chromium bipolar head system (Co–Cr universal head bipolar system and titanium Accolade II stem, Stryker Orthopedics, MI, USA) (Figure 1(a)). Six months after surgery, the patient complained of gradually increasing pain in right hip, but patient did not consult our hospital until 2 years after the initial surgery. The right hip pain had progressed and patient could not walk for more than 20 minutes. Examination revealed a large erythema of the skin and a localized heat sensation from the buttocks to the lateral thigh (Figure 2(a)) as well as many swollen lymph nodes in the inguinal region. There was tenderness in the right scarpa delta and inguinal lymphadenopathy. Laboratory data revealed a normal WBC count, C-reactive protein (CRP) of 1.3 mg/dl, anti-cyclic citrullinated peptide antibody of under 0.5 U/ml, rheumatoid factor of under 3 IU/ml, and negative in antinuclear antibody. A plain radiograph of the pelvis showed a loss of hip joint space, but no evidence of femoral stem loosening (Figure 1(b)). Magnetic resonance imaging (MRI) of the pelvis revealed a large annular lesion distended around the greater trochanter on T1-weighted images and many small, honeycomb-shaped bodies within the cystic lesion on the T2-weighted STIR sequence (Figure 3(a)). Numerous inguinal lymphadenopathies were also recognized on the T2-weighted STIR sequence (Figure 3(b)). Based on typical clinical symptoms and medical history, metal hypersensitivity was suspected, and a patch test was performed. The metal patch test was based on the 48-hour closed patch test in which a tape with a reagent was applied to the patient's back and assessed by a dermatologist. Assessments were made every 48 hours, 3 days, and 7 days. However, the patch test was negative for nickel (Ni), cobalt (Co), chromium (Cr), zinc (Zn), gold (Au), silver (Ag), platinum (Pt), and palladium (Pd). In addition, an in vitro lymphocyte transformation test was performed but proved negative for any traces of titanium (Ti), oxidized Ti, Ni, Co, Cr, Zn, Au, Ag, and aluminum (Al). Because of the mildly elevated CRP value, a deep infection could not be ruled out, and a two-stage revision surgery was scheduled. In the first surgery, there was no obvious drainage, no loosening of the implant, and no pseudotumor, but the joint cavity was filled with rice bodies (Figure 4(a)). Because of the potential for infection, an antibiotic cement spacer was inserted at the time of the first surgery (Figure 5(a)). Bacteria culture tests were performed on the rice body, synovial membrane, and joint fluid collected during surgery for 72 hours each, and no bacteria were detected in any of the samples. The pathological diagnosis of the rice body was a rice-body-containing cyst showing fibrin precipitation due to nonspecific chronic synovitis. No neutrophil infiltration associated with infection or metallic wear debris was observed (Figure 4(b)). Since no sign of infection was found, a second surgery was performed with a cemented THA (Rim fit cup and Exeter, Stryker Orthopedics, MI, USA) and ceramic head (BIOLOX delta V40 Ceramic Head, Stryker Orthopedics, MI, USA) 2 weeks after the first surgery (Figure 5(b)). Postoperatively, hip pain, inguinal lymphadenopathy, and the skin erythema disappeared (Figure 1(b)), and the modified Harris hip score 3 years after surgery was 100 points, with no recurrence of erythema and satisfactory results.

## 3. Discussion

A reliable method of diagnosing metal hypersensitivity to orthopedic metal implants has not yet been established. There are also reports that preoperative screening should be performed in patients with a history of metal hypersensitivity [7–10]. Generally, the patch and the lymphocyte transformation tests are often used to assess allergic reactivity. In the present case, the patient had a history of hay fever, mild atopic dermatitis, and a skin allergy to metal jewelry prior to surgery, and clinical symptoms of refractory erythema, swollen inguinal lymph nodes, and unexplained hip pain were observed after BHA. Based on these medical histories and clinical symptoms, metal hypersensitivity was suspected, but the tests recommended for diagnosis, such as the patch test and the lymphocyte transformation test were negative. Therefore, a definitive diagnosis was not reached. On the other hand, several papers describe that patch tests are subjective in evaluation and have reported cases where tests have yielded false positives [9, 11, 12]. In addition, intradermal Langerhans cells mediate the immune response elicited by the patch test; whereas, the metal hypersensitivity related to arthroplasty in the joint space is mediated by lymphocytes and macrophages. As a result, it can be concluded that the patch test is immunologically different from the metal hypersensitivity associated with arthroplasty [13]. It has been reported that the lymphocyte transformation test is relatively useful [14, 15]. However, there is no consensus on the specificity of lymphocyte transformation tests [13, 15, 16]. According to a survey conducted by the European Contact Dermatitis Society and the American Contact Dermatitis Society, there are four major criteria for diagnosing eruptions due to metal hypersensitivity in dermatology [17]. Major criteria were as follows:

1. Chronic dermatitis beginning weeks to months after metallic implantation.

2. Eruption overlying the metal implant.

3. Positive patch test.

4. Complete clearing after removal of the potentially allergenic implant.

In the present case, three of the four items, with the exception of the positive patch test, met the major criteria. A characteristic finding was the dramatic disappearance of the chronic eruption around the surgical site soon after revision surgery in which Co–Cr containing BHA was replaced by cemented THA. Unfortunately, no inguinal lymph node biopsy was performed during surgery, so a histological diagnosis of the swollen lymph nodes could not be made. Many rice bodies present in the joint were suspected to be osteochondromatosis based on MRI findings and macroscopic appearance, but the pathological diagnosis was rice bodies containing fibrin. Fibrin deposition in synovial tissue may be the result of some chronic inflammation of the hip joint. However, it cannot be proven that the chronic inflammation of the hip joint was triggered by metal hypersensitivity. On the other hand, there were no histological findings that were characteristic of metallosis or a pseudotumor caused by metal wear. No findings of metal hypersensitivity were observed in either in vivo or in vitro preoperative testing, but to ensure absolute reliability, an implant containing Co and Cr was avoided during revision surgery. Revision THA using a cemented implant and a ceramic head was performed as polyethylene (PE) and polymethylmethacrylate (PMMA) particles are relatively large and less likely to cause hypersensitivity reactions. In patients with suspected metal hypersensitivity who underwent revision THA and total knee arthroplasty (TKA) for a well-fixed implant, there have been reports of symptom improvement after the initial implant was replaced with a hypoallergenic metal implant. Thakur et al. reported that unexpected persistent knee pain after TKA had improved in six knees of five patients in which implants containing Co–Cr were replaced with Oxinium and Ti [18]. They also reported that two out of five patients had a negative preoperative patch test. Campbell et al. reported symptom improvement in four cases in which metal-on-metal resurfacing THA containing Co–Cr was replaced with ceramic-on-ceramic THA [19]. In general, hypoallergenic implants such as Oxinium, titanium, and ceramic are recommended in cases of suspected metal hypersensitivity [20, 21]. In the present case, a definitive diagnosis could not be made. We decided on a treatment plan based on the diagnosis of exclusion. After ruling out infection by bacterial culture and the exclusion of metal allergy by both in vivo and in vitro testing, we chose the treatment that presented the least risk. However, excellent results were obtained by changing the Co–Cr bipolar head and titanium stem to a cemented THA and ceramic head. Revision THA with a cemented THA and ceramic head is considered to be one of the options for patients with suspected metal hypersensitivity.

There are limitations in this case report. For example, we were unable to test all metals associated with the prosthesis due to the cost of testing. However, manganese (Mn) is present in trace amounts in Co–Cr alloys and should have originally been tested. Furthermore, regarding using PMMA, we also determined that hypersensitivity reactions are unlikely, while some papers suggest the possibility of PMMA-induced hypersensitivity after arthroplasty. In the future, when selecting cement THA for patients with suspected metal hypersensitivity, the lymphocyte transformation test should also be performed for PMMA.

## 4. Conclusion

In this case, metal hypersensitivity after BHA was suspected based on the patient's medical history, but preoperative screening was negative. Conversion to cemented THA was performed, and there has been no recurrence of erythema 3 years after surgery. Patients with clinically suspected metal hypersensitivity and negative preoperative screening should undergo THA and revision THA using hypoallergenic implants.

## Data Availability

The datasets analyzed during the current study are available from the corresponding author.

## Abbreviations

THA: Total hip arthroplasty
BHA: Bipolar hemiarthroplasty
WBC: White blood cell
MRI: Magnetic resonance imaging
Ni: Nickel
Co: Cobalt
Cr: Chromium
Zn: Zinc
Au: Gold
Ag: Silver
Pt: Platinum
Pd: Palladium
Ti: Titanium
Al: Aluminum
CRP: C-reactive protein
PE: Polyethylene
PMMA: Polymethylmethacrylate
TKA: Total knee arthroplasty.
